# TLR4 induces tumor growth and inhibits paclitaxel activity in MyD88-positive human ovarian carcinoma *in vitro*

**DOI:** 10.3892/ol.2013.1759

**Published:** 2013-12-16

**Authors:** AN-CONG WANG, YUE-BING MA, FENG-XIA WU, ZHI-FANG MA, NAI-FU LIU, RONG GAO, YONG-SHENG GAO, XIU-GUI SHENG

**Affiliations:** 1 Department of Obstetrics and Gynecology, Linyi People’s Hospital, Linyi, Shandong 276002, P.R. China; 2 Department of Gynecologic Oncology, Shandong Cancer Hospital and Institute, Jinan, Shandong 250117, P.R. China; 3 Department of Anatomy, Shandong University, Jinan, Shandong 250012, P.R. China; 4 Department of Pathology, Shandong Cancer Hospital and Institute, Jinan, Shandong 250117, P.R. China

**Keywords:** ovarian cancer, TLR4, MyD88, paclitaxel, chemotherapy

## Abstract

In ovarian cancer patients, chemotherapy resistance is the principal factor restricting long-term treatment. Paclitaxel (Pac) has been previously reported to be a ligand to Toll-like receptor 4 (TLR4). It was determined that TLR4 signaling is divided into the following two pathways: Myeloid differentiation factor 88 (MyD88)-dependent and MyD88-independent. The present study investigated the effect of TLR4 ligation by Pac in MyD88-positive (MyD88^+^) and MyD88-negative (MyD88^−^) human ovarian cancer cell lines. An RNA interference expression vector was specifically constructed to target TLR4 mRNA, which was stably transfected into the human ovarian cancer cell lines (SKOV3, OVCAR3, A2780 and 3AO). Cytokines, including interleukin (IL)-6 and IL-8, were detected. Cell proliferation and apoptosis were assessed in the cells transfected with scramble control and TLR4 shRNA to explore the possible functions of TLR4 in ovarian cancer cell growth. It was found that lipopolysaccharide and Pac significantly increase the secretion of IL-6 and IL-8 in the SKOV3 cell line. Similarly, Pac resulted in a significant upregulation of IL-6 and IL-8 in OVCAR3 cells, but not in A2780 and 3AO cells. These results suggested that in MyD88^+^ ovarian cancer cell lines, TLR4 depletion shows increased sensitivity to Pac treatment in inhibiting cell proliferation compared with in cells without TLR4 knockdown. On the contrary, such changes were not found in MyD88^−^ cells (A2780 and 3AO). TLR4 negatively regulates Pac chemotherapy, particularly in terms of cell proliferation, and TLR4 may be a novel treatment target in Pac-resistant ovarian cancer.

## Introduction

Toll-like receptors (TLRs) are a family of pattern recognition receptors. To date, 11 human TLRs and 13 mouse TLRs have been identified (1). Mammalian TLRs recognize microbial products and initiate innate immune responses (2).

Although a limited number of studies have analyzed the correlation between TLR expression and human malignancy, several studies concerning the expression of TLRs and cancer have been conducted (3).

The Toll-related proteins were first identified in mammals (4) and the mammalian TLR4 was rapidly demonstrated to be responsible for the recognition of lipopolysaccharide (LPS) (5). It has been previously reported that TLR4 negatively regulates the Salmonella-induced antitumor activity (6). Notably, TLR4 has been reported to be important in promoting the immune escape of human lung cancer cells by inducing immunosuppressive cytokines and apoptosis resistance (7).

In ovarian cancer patients, chemotherapy resistance is the principal factor restricting long-term treatment (8). Previously, paclitaxel (Pac) has been reported to be a ligand to TLR4 (9). The current study investigated the effects of TLR4 in ovarian cancer, particularly in Pac chemotherapy. Myeloid differentiation factor 88 (MyD88) was originally isolated as a gene that is induced rapidly during the interleukin (IL)-6-stimulated differentiation of M1 myeloleukemic cells into macrophages (10). IL-6 is considered to be involved in host immune responses to types of ovarian cancer (11,12). IL-6 has also been demonstrated to provide paracrine growth stimulation when monocytes are attracted to types of ovarian cancer that produce macrophage colony-stimulating factor (13). IL-6 signaling in ovarian cancer cells regulates tumor cell proliferation, invasion and angiogenesis (14,15) and IL-8 has also been reported to promote ovarian tumor growth *in vivo* (16). Previously, it has been reported that TLR4 signaling is divided into the following two pathways: MyD88-dependent and MyD88-independent (17,18). A correlation between MyD88 expression and patients’ progression-free survival has shown that patients whose tumors do not express MyD88 exhibit a significantly improved progression-free interval compared with patients whose tumors express high levels of MyD88 (19).

The present study investigated the role of TLR4 in ovarian cancer cells and the effect of TLR4 ligand by Pac in MyD88^+^ and MyD88^−^ human ovarian carcinoma *in vitro*.

## Materials and methods

### Reagents

Pac was purchased from Sigma-Aldrich (St. Louis, MO, USA) and the rabbit polyclonal antibodies against TLR4 and MyD88 were purchased from Santa Cruz Biotechnology, Inc., (Santa Cruz, CA, USA).

### Cell culture and samples

The *in vitro* experiments were performed with human ovarian cancer cell lines, SKOV3, OVCAR3, A2780 and 3AO. All the cell lines were obtained from the Basic Research Center, Shandong Cancer Hospital and Institute (Jinan, China). Cells were cultured in RPMI-1640 medium (Gibco-BRL, Carlsbad, CA, USA) supplemented with 10% heat-inactivated fetal bovine serum (FBS; Gibco-BRL) and incubated under standardized conditions (37°C; 5% CO_2_ atmosphere).

Samples of normal ovarian tissue adjacent to tumor (n=12) and borderline (n=8) and malignant (n=24) tumors were collected with the approval of the Ethics Committee of the Shandong Cancer Hospital and Institute.

### RNA isolation and reverse transcription-polymerase chain reaction (RT-PCR)

Total RNA was extracted using the TRIzol reagent kit (Invitrogen Life Technologies, Carlsbad, CA, USA) according to the manufacturer’s instructions. Reverse transcription was performed using SYBR ExScript RT-PCR kit (Takara Bio, Inc., Shiga, Japan). The following set of primers were used for amplification: i) human TLR4 sense, 5′-AATGGATCAAGGACCAGAGG-3′ and antisense, 5′-CAGCCAGCAAGAAGCATCAG-3′; and ii) human MyD88 sense, 5′-CGCCGGATGGTGGTGGTTGT-3′ and antisense, 5′-TGTAGTCGCAGACAGTGATGAACC-3′. The primers were used to amplify an 197-bp fragment of TLR4 cDNA and an 186-bp fragment of MyD88 cDNA. The following primers were used for β-actin: forward, 5′-TTGTATCGTGGAAGGACTCA-3′ and reverse, 5′-TGTCATCATATTTGGCAGGTTT-3′. TLR4 was used to amplify a 197-bp fragment. Forty cycles of PCR were performed at 95°C for 30 sec, 63°C for 30 sec and 72°C for 45 sec. The primers for human MyD88 were used to amplify a 186-bp fragment of MyD88 cDNA. Thirty cycles of PCR were performed at 95°C for 30 sec, 61°C for 30 sec and 72°C for 45 sec.

### SDS-PAGE and western blot analysis

Protein was denatured in sample buffer [2.5% SDS, 10% glycerol, 5% β-mercaptoethanol, 0.15 mol/l Tris (pH 6.8) and 0.01% bromophenol blue] and subjected to 10–12% SDS-PAGE as previously described (6). The following antibodies were used: Rabbit anti-TLR4 (1:1,000), -MyD88 (1:1,000) and -actin (1:10,000) (Santa Cruz Biotechnology, Inc.). Signals were detected using ECL western blotting detection reagents (Pierce Biotechnology, Inc., Rockford, IL, USA) according to the manufacturer’s instructions.

### Immunohistochemistry

Paraffin sections of tumor tissues were deparaffinized and microwaved while immersed in 0.01 M citrate buffer (pH 6.0) for 20 min. Sections were washed with PBS and incubated overnight at 4°C with polyclonal rabbit anti-human TLR4 antibody (1:50) or with polyclonal rabbit anti-human MyD88 (1:50; Santa Cruz Biotechnology, Inc.). Following washing, tissues were incubated with horseradish peroxidase-labeled anti-rabbit antibody for 1 h, followed by 3,3′-diaminobenzidine (Dako, Carpinteria, CA, USA). The results for TLR4 and MyD88 expression in tissues were scored by two independent investigators based on the following levels of staining intensity: None (−), weak (+), moderate (++), or strong (+++).

### Incubation of tumor cells with TLR4 ligands

In all experiments testing the effects of LPS or Pac on tumor cells, LPS was used at a concentration of 10 μg/ml and Pac at 2 μM.

### Cytokine and chemokine production

Cytokine and chemokine production by the human ovarian cancer cells was determined using a Luminex-100 System (Luminex, Austin, TX, USA). Supernatants of tumor cells seeded in 12-well plates at 5×10^5^ cells/well in 1 ml of LPS or Pac medium were collected following 36 h of incubation. The levels of IL-6 and IL-8 were measured using panels of capture antibody-coated beads and the labeled detection antibodies, which were pretested and qualified by the manufacturer to ensure the absence of cross-reactivity. The assay sensitivity varied between 5 and 15 pg/ml.

### Caspase-Glo 3/7 assay

In total, 10 μg of protein in a 50 μl total volume was mixed with 50 μl equilibrated Caspase-Glo 3/7 reagents (Promega Corporation, Madison, WI, USA). Following incubation at room temperature for 1 h, luminescence was measured using TD 20/20 luminometer (Turner Designs, Inc., Sunnyvale, CA, USA). Blank values were subtracted and fold increase in activity was calculated based on the activity measured from untreated cells. Each sample was measured in triplicate.

### Construction of RNA interference targeting the TLR4 gene in the vector

Small interfering RNA (siRNA) oligonucleotides specifically targeting TLR4 oligonucleotides were confirmed to be valid by the authors and were designed with the following sequences: Sense, 5′-GGTAAGGAATGAGCTAGTA-3′ and antisense, 5′-TACTAGCTCATTCCTTACC-3′. The pGenesil-1 negative control vector (Wuhan Genesil Biotechnology Co., Ltd., Wuhan, China) was used as the negative control plasmid in all experiments as previously described (20).

### Transcription and production of stable clones

The day prior to transfection, cells were trypsinized and plated at a density of 5×10^5^ cells/well in six-well tissue culture plates (Corning Inc., Corning, NY, USA). Cells were rinsed twice with serum-free RPMI-1640 when the density reached >90% confluence. Next, pGenesil-shTLR4 (RNA interference expression vectors) or pGenesil-shControl (control vector) and Lipofectamine 2000 mixtures, prepared in OptiMEM (Invitrogen Life Technologies), were added. Following 5 h of incubation, the plasmid-Lipofectamine 2000 mixture was removed and RMPI-1640 plus 10% FBS and 800 μg/ml geneticin (Invitrogen Life Technologies) for SKOV3 and OVCAR3 cells, 500 μg/ml G418 for 3AO cells and 600 μg/ml geneticin for A2780 cells were added. All the non-transfected cells died within 7 days and a number of surviving transfected cells were harvested 21 days later.

### MTT assay

Stable clone cells were seeded into 96-well culture plates (5,000/well) and treated with 2 μmol/l Pac (2 μM) for 24 h. As the control for normal cell proliferation, 0.1% ethanol was used. At the end of each treatment, cells were incubated as previously described (21).

### Statistical analysis

Data are presented as the mean ± standard deviation for continuous variables, and the frequency and percentage for categorical variables. Results were statistically evaluated by analysis of variance. SPSS 17.0 software for Windows was used for statistical treatment (SPSS, Inc., Chicago, IL, USA). P<0.05 was considered to indicate a statistically significant difference.

## Results

### Expression of TLR4 and MyD88 in ovarian cancer tissues

Expression of TLR4 and MyD88 were assessed *in situ* on paraffin sections of normal ovarian tissue adjacent to tumor (n=12) and borderline (n=8) and malignant (n=24) tumors. TLR4 was found to exhibit moderate (++) or strong (+++) expression in malignant (20/24) and borderline (5/8) tumors and normal ovarian epithelium (6/12). However, the expression of MyD88 in malignant tumors was considerably greater (18/24) than that in normal ovarian tissue (1/8) or in borderline tumors (1/12) (Table I).

### Expression of TLR4 and MyD88 signaling adapter protein in epithelial ovarian cancer (EOC) cell lines

**Figure 1:**
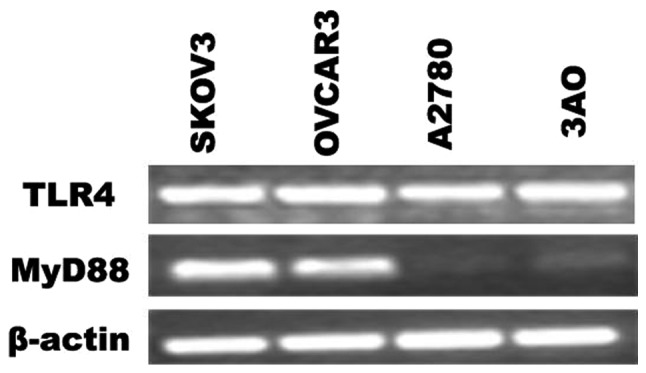
Expression of TLR4 and MyD88 in EOC cells at the mRNA level. TLR4 was expressed in all EOC cell lines; however, positive expression of MyD88 was identified in SKOV3 and OVCAR3 cells, while negative MyD88 expression was observed in A2780 and 3AO cells. TLR4, Toll-like receptor 4; MyD88, myeloid differentiation factor 88; EOC, epithelial ovarian cancer.

Furthermore, the expression of TLR4 and MyD88 in EOC cells was evaluated by RT-PCR. As shown in Fig. 1, the mRNA of TLR4 were expressed in EOC cell lines. However, the expression of MyD88 was found to differ; SKOV3 and OVCAR3 cells were MyD88-positive, while A2780 and 3AO cells were MyD88-negative.

### LPS- and Pac-induced cytokine production in MyD88^+^ cells

**Figure 2:**
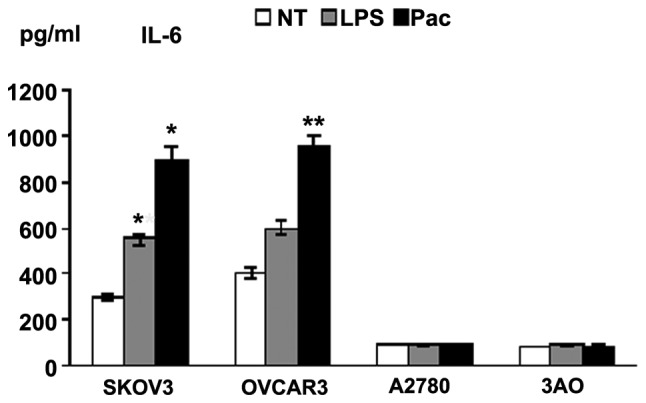
Levels of cytokines in tumor cell SNs. SNs were collected from tumor cells cultured at a density of 5×10^5^ cells/well following 36 h of treatment and tested for levels of IL-6. Three independent experiments were performed. ^*^P<0.05, vs. NT/SKOV3 and ^**^P<0.05, vs. NT/OVCAR3. SNs, supernatants; IL, interleukin; LPS, lipopolysaccharide; Pac, paclitaxel. NT, cells not treated with LPS or Pac.

**Figure 3:**
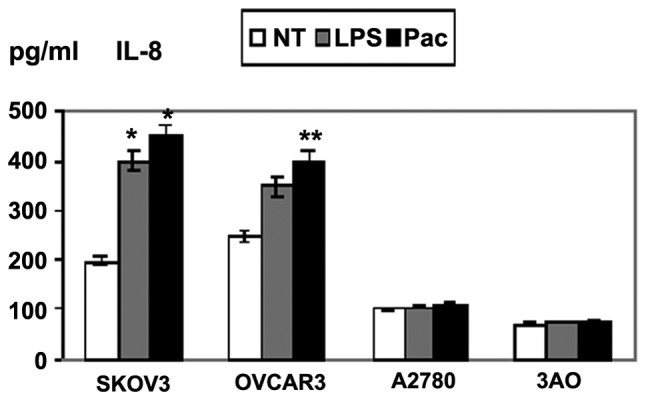
Levels of cytokines in tumor cell SNs. SNs were collected from tumor cells cultured at a density of 5×10^5^ cells/well following 36 h of treatment and tested for levels of IL-8. Three independent experiments were performed. ^*^P<0.05, vs. NT/SKOV3 and ^**^P<0.05, vs. NT/OVCAR3. SNs, supernatants; IL, interleukin; LPS, lipopolysaccharide; Pac, paclitaxel. NT, cells not treated with LPS or Pac.

Supernatants of the four cell lines exposed to LPS or Pac for 36 h were analyzed for levels of inflammatory cytokines and growth factors. SKOV3 and OVCAR3 cells constitutively secreted a wide range of cytokines and chemokines, including IL-6 and IL-8. By contrast, A2780 and 3AO cells produced low levels of these cytokines. LPS and Pac significantly increased the secretion of IL-6 and IL-8 in SKOV3 cells. Similarly, Pac resulted in a significant upregulation of IL-6 and IL-8 in OVCAR3 cells, but not in A2780 and 3AO cells (Figs. 2 and 3).

### Effect of shRNA on TLR4 gene expression

**Figure 4:**
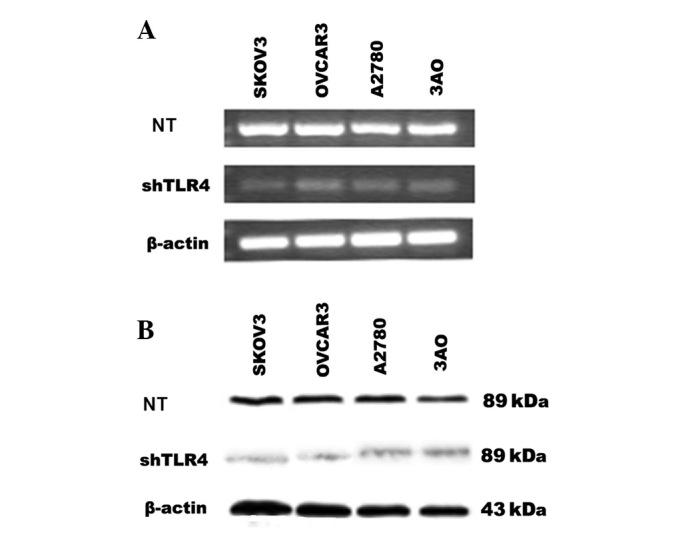
Effect of TLR4 knockdown in EOC cells using RT-PCR and western blot analysis. (A) Evaluation of mRNA expression of TLR4 in SKOV3, OVCAR3, A2780 and 3AO cell lines. (B) Western blot analysis for TLR4 protein expression in the four cell lines. shTLR4 cells were not treated with TLR4 interference vector transfection. TLR4, Toll-like receptor 4; EOC, epithelial ovarian cancer; RT-PCR, reverse transcription-polymerase chain reaction; NT, cells not treated with lipopolysaccharide or paclitaxel.

To investigate the potential role of TLR4 in EOC cell lines, pGenesil-shTLR4 directed at nucleotides 2,202–2,220 of TLR4 was used to selectively reduce TLR4 gene expression in EOC cell lines, SKOV3, OVCAR3, A2780 and 3AO. As shown in Fig. 4, the shTLR4 significantly reduced the expression of the TLR4 mRNA and protein in these cells (SKOV3/shTLR4, OVCAR3/shTLR4, A2780/shTLR4 and 3AO/shTLR4). The control-scrambled sequence, shControl, exhibited no effect on TLR4 gene expression in cells (SKOV3/shControl, OVCAR3/shControl, A2780/shControl and 3AO/shControl). Thus, the application of TLR4-directed shTLR4 was an effective and selective method of long-term suppression of endogenous TLR4 levels, making it possible to determine in experiments the role of endogeneous TLR4 on EOC cells.

### Knockdown of TLR4 depressed cytokine production in MyD88^+^ cells

**Figure 5:**
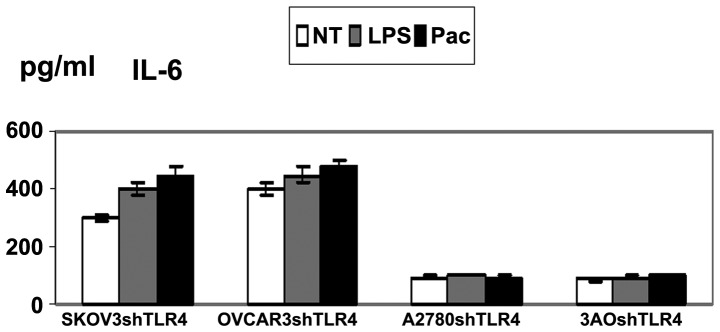
Levels of cytokines in tumor cell SNs. SNs were collected from tumor cells cultured at a density of 5×10^5^ cells/well following 36 h of treatment and tested for levels of IL-6. SNs, supernatants; IL, interleukin; LPS, lipopolysaccharide; Pac, paclitaxel. NT, cells not treated with LPS or Pac.

**Figure 6:**
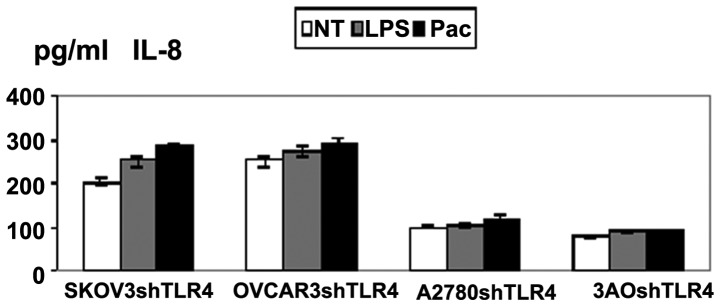
Levels of cytokines in tumor cell SNs. SNs were collected from tumor cells cultured at a density of 5×10^5^ cells/well following 36 h of treatment and tested for levels of IL-8. SNs, supernatants; IL, interleukin; LPS, lipopolysaccharide; Pac, paclitaxel. NT, cells not treated with LPS or Pac.

Supernatants of the four cell lines exposed to LPS or Pac for 36 h were analyzed for levels of inflammatory cytokines and growth factors. SKOV3/shTLR4 and OVCAR3/shTLR4 cells were found to secrete low levels of cytokines IL-6 and IL-8. However, these changes were not found in A2780/shTLR4 and 3AO/shTLR4. These results suggested that LPS/Pac results in a significant downregulation of IL-6 and IL-8 in MyD88^+^, but not in MyD88^−^ cells, in which TLR4 had been knocked down (Figs. 5 and 6).

### Effect of TLR4 on the Pac sensitivity of MyD88^+^ EOC cells

**Figure 7:**
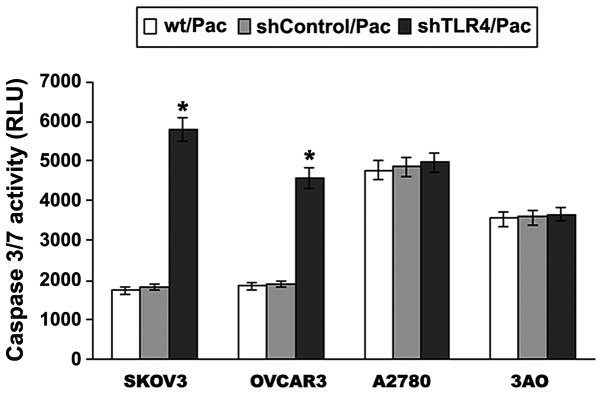
Following TLR4 knockdown, the apoptotic response of Pac (2 μM) treatment in MyD88^+^ and MyD88^−^ EOC cell lines was investigated. EOC cell lines were treated with 2 μmol/l Pac for 24 h and the levels of caspase-3/7 were measured using the Caspase-Glo 3/7 assay. Data are presented as the mean ± SD from at least three independent experiments. A significant increase was identified in caspase-3/7 activity following Pac treatment in SKOV3/shTLR4 cells compared with SKOV3 and SKOV3/shControl cells, as was the case with the OVCAR3 cells. ^*^P<0.001, vs. wt/Pac and shControl/Pac. TLR4, Toll-like receptor 4; MyD88, myeloid differentiation factor 88; EOC, epithelial ovarian cancer; Pac, paclitaxel; wt/Pac, SKOV3/OVCAR3/A2780/3AO parental cells treated with Pac; shControl/Pac, SKOV3/OVCAR3/A2780/3AO shControl cells treated with Pac; shTLR4/Pac, SKOV3/OVCAR3/A2780/3AO shTLR4 cells treated with Pac.

Of note, a positive increase in caspase-3/7 activity was identified between the MyD88^+^ and MyD88^−^ cell lines (Fig. 7). In order to identify whether the MyD88^+^ cells respond to cell apoptosis through TLR4-MyD88 signaling, RNA interference was used to knock down the expression of TLR4 and the results are shown in Fig. 7. A significant increase was identified in caspase-3/7 activity following Pac treatment in SKOV3/shTLR4 cells compared with SKOV3/shControl cells (P<0.001), as was the case with OVCAR3 cells (P=0.000). No significant difference was observed in changes of caspase-3/7 activity between A2780/shTLR4 and A2780/shControl cells, as well as with 3AO cells. The results suggested that the TLR4-MyD88 signaling negatively regulates ovarian cancer cell sensitivity to Pac.

### Knockdown of TLR4 depressed cell proliferation in MyD88^+^ EOC cells

**Figure 8:**
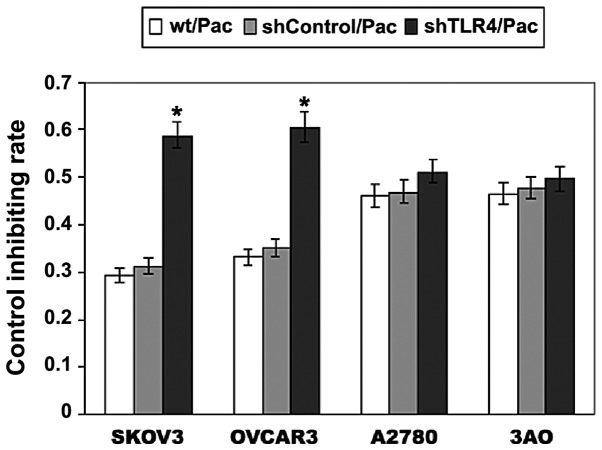
Effect of TLR4 knockdown on EOC cell growth using MTT assay. SKOV3/shTLR4 cells were inhibited by ~60% in the presence of Pac at 2 μmol/l compared with parental SKOV3 (29%) and SKOV3/shControl (31%) cells. OVCAR3/shTLR4 cells showed a marked change compared with parental OVCAR3 and OVCAR3/shControl cells. However, no significant changes were identified among the parental, shControl and shTLR4 cells in the A2780 and 3AO cell lines. ^*^P<0.001, vs. wt/Pac and shControl/Pac. TLR4, Toll-like receptor 4; EOC, epithelial ovarian cancer; Pac, paclitaxel; wt/Pac, SKOV3/OVCAR3/A2780/3AO parental cells treated with Pac; shControl/Pac, SKOV3/OVCAR3/A2780/3AO shControl cells treated with Pac; shTLR4/Pac, SKOV3/OVCAR3/A2780/3AO shTLR4 cells treated with Pac.

The effect of Pac on cell proliferation in SKOV3, OVCAR3, A2780 and 3AO cell lines was investigated. In Fig. 8, the growth inhibiting rate (GIR) of SKOV3/shTLR4 cells was ~60% following 24 h treatment of 2 μM Pac, which is significantly higher than those of the parental SKOV3 (29%) and SKOV3/shControl (31%) cells (P<0.001). In the OVCAR3 cell line, the same treatments were used as with the parental OVCAR3, OVCAR3/shControl and OVCAR3/shTLR4. The GIR was ~61% in OVCAR3/shTLR4, which was higher than that of the parental OVCAR3 (33%) and OVCAR3/shControl (35%) (P<0.001). However, no difference was observed in the proliferation among the three types of A2780 cells (parental A2780, A2780/shControl and A2780/shTLR4), as well as in the 3AO cells (parental A2780, A2780/shControl and A2780/shTLR4). These results demonstrated that knockdown of TLR4 significantly restores the sensitivity of Pac in MyD88^+^ ovarian cancer cells.

## Discussion

The successful treatment of ovarian cancer remains a major challenge. Overall, >85% patients presenting with advanced disease are likely to relapse. Recurrence defines incurable disease in the majority of cases. The main obstacle to effective treatment is the failure of initial therapy to eradicate a sufficient number of tumor cells to prevent disease recurrence. Pac is a product of the Pacific yew and its antimitotic actions are due to its ability to bind and stabilize microtubules, which prevent accurate cell division during mitosis (22,23). Pac induces the secretion of inflammatory cytokines in murine macrophages in a TLR4-dependent manner in addition to its antitumor effects. Although, the effect of Pac on human macrophages is controversial (24). Few previous studies have analyzed the contribution of innate immunity pathways to the mechanism of action of Pac (25). Pac is a first-line chemotherapeutic agent used in the treatment of EOC as well as recurrent EOC (26), and is known to be a TLR4 ligand (27,28). In the present study, it was identified that Pac activates TLR4 signaling, which increases ovarian cancer cell proliferation.

Previously, MyD88 has been reported to be a negative regulator of TLR signaling (29,30,31). It has been identified that MyD88 is an essential downstream component of the TLR4 signaling cascade mediating Pac resistance (19). In addition, it has been reported that LPS-stimulated tumor cell supernatants inhibit T cell proliferation and natural killer cell activity. Blockade of the TLR4 pathway reverses the functions of these cells *in vitro* and *in vivo*, delays tumor growth and, thus, prolongs the survival of tumor-bearing mice (32). In the present study, TLR4 was found to exhibit moderate (++) or strong (+++) expression in malignant (20/24) and borderline (5/8) tumors and normal ovarian epithelium (6/12). The expression of MyD88 in malignant tumors was considerably greater (18/24) than that in normal ovarian tissue (1/8) or borderline tumors (1/12). The results of the present study support that MyD88 acts as a downstream factor and combines with TLR4 to increase the proliferation of ovarian cancer cells.

If TLR4 signaling highlights a survival benefit to tumor cells and alters their sensitivity to Pac, then its silencing via siRNA must aid in identifying the molecular mechanisms responsible for LPS- and Pac-mediated effects. We hypothesized that in TLR4-MyD88 signaling, TLR4 is activated by Pac. MyD88^+^ human ovarian carcinoma cells (SKOV3 and OVCAR3) and MyD88^−^ ovarian carcinoma cell lines (A2780 and 3AO) were selected to investigate the TLR4 effects on apoptosis with Pac chemotherapy. The molecular mechanisms of chemotherapy resistance are considered to be associated with apoptosis inhibition (33,34). Acquired resistance to chemotherapy is a significant impediment to effective cancer therapy (35). Notably, silencing of TLR4 expression in MyD88^+^ EOC cells results in sensitization of the cells to Pac-induced apoptosis and this sensitization is accompanied by the inhibition of cytokine IL-6 and IL-8 production in response to Pac and LPS. In the current study, MyD88^+^ cells (SKOV3 and OVCAR3) constitutively secreted a wide range of cytokines including, IL-6 and IL-8. By contrast, MyD88^−^ cells (A2780 and 3AO) produced low levels of these cytokines. LPS and Pac significantly increased the secretion of IL-6 and IL-8 in SKOV3 and OVCAR3 cells, but not in A2780 and 3AO cells (Figs. 2 and 3).

The present study used RNA interference to knock down the expression of TLR4 in SKOV3, OVCAR3, A2780 and 3AO cell lines. Cytokine production in response to LPS and Pac stimulation was significantly inhibited in SKOV3/shTLR4 and OVCAR3/shTLR4 cells. No changes in cytokine production were observed in A2780/shTLR4 and 3AO/shTLR4 cells (Figs. 5 and 6).

Caspase-Glo 3/7 assay was used to investigate the apoptosis of ovarian cancer cells. When TLR4 was knocked down, a positive increase in caspase-3/7 activity was identified following Pac treatment between MyD88^+^ and MyD88^−^ cell lines. The mechanism responsible for Pac resistance in ovarian cancer is not completely understood. The present study confirmed that there is a negative correlation between MyD88 expression and Pac-induced apoptosis in TLR4 signaling, consistent with the results of a previous study (36).

In addition, RNA interference was used to knockdown the expression of TLR4 in SKOV3, OVCAR3, A2780 and 3AO cell lines. In the caspase-Glo 3/7 assay, a significant increase of caspase-3/7 activity was identified in SKOV3/shTLR4 and in OVCAR3/shTLR4 cells (Fig. 7).

The results of the current study indicated that TLR4-MyD88 signaling negatively regulates Pac treatment. Knockdown of TLR4 may increase Pac chemosensitivity in MyD88^+^ cells. In addition to the caspase-Gol 3/7 assay, cell proliferation was evaluated and a significant increase of GIR was identified in SKOV3/shTLR4 and OVCAR3/shTLR4 cell lines. However, in A2780/shTLR4 and 3AO/shTLR4 cells, no significant changes were observed compared with their control cells (Fig. 8). The present study revealed that the proliferation and survival of the cancer cells is regulated by a specific defense mechanism in TLR4/MyD88 signaling.

In conclusion, the observations of the current study imply that Pac activates TLR4-MyD88 signaling, which increases ovarian cancer cell proliferation. Although the results suggest that the knockdown of TLR4 inhibits cell growth and that IL-6 and IL-8 levels are associated with MyD88 (+) EOC cells, the precise underlying molecular mechanisms responsible for these observations remain to be established. As TLR4 is functionally associated with tumor progression, TLR4 is likely to be a promising target for tumor therapy in the future.

## Figures and Tables

**Table I:** Results for TLR4 and MyD88 expression in tissues.

Definite histology	TLR4	MyD88
Malignant tumor cases		
1	++	−
2	+++	++
3	+++	++
4	++	−
5	+++	+++
6	++	+++
7	+++	++
8	+	+++
9	+++	++
10	++	−
11	++	++
12	+++	++
13	+++	+++
14	+++	++
15	++	+
16	++	++
17	++	+
18	+	++
19	++	++
20	++	+++
21	+	+
22	++	++
23	+	++
24	++	+++
Borderline cases		
1	++	−
2	++	+
3	+	−
4	+++	++
5	++	+
6	+	−
7	++	+
8	+	+
Normal ovarian epithelium cases		
1	++	+
2	+++	++
3	++	+
4	++	+
5	+	+
6	+	−
7	++	+
8	+	+
9	+	−
10	+	+
11	++	+
12	+	+
P-value	0.022^a^	0.001^b^

Summary of the pathological characteristics of tissues. TLR4 and MyD88 expression were scored by the following levels of staining intensity: none (−); weak (+); moderate (++); or strong (+++).

a P=0.022, vs. the proportion of TLR4-positive expression in normal ovarian epithelium and

b P=0.001, vs. MyD88-positive expression in borderline and normal ovarian epithelium.

TLR4, Toll-like receptor 4; MyD88, myeloid differentiation factor 88.

## Abbreviations

TLR4: Toll-like receptor 4
Pac: paclitaxel
MyD88: myeloid differentiation factor 88
